# Breath-holding as a novel approach to risk stratification in COVID-19

**DOI:** 10.1186/s13054-021-03630-5

**Published:** 2021-06-14

**Authors:** Ludovico Messineo, Elisa Perger, Luciano Corda, Simon A. Joosten, Francesco Fanfulla, Leonardo Pedroni, Philip I. Terrill, Carolina Lombardi, Andrew Wellman, Garun S. Hamilton, Atul Malhotra, Guido Vailati, Gianfranco Parati, Scott A. Sands

**Affiliations:** 1 Division of Sleep and Circadian Disorders, Departments of Medicine and Neurology, Brigham & Women’s Hospital & Harvard Medical School, Boston, MA USA; 2 Adelaide Institute for Sleep Health (AISH), Flinders Health and Medical Research Institute (FHMRI), Flinders University, 5 Laffer Drive, Bedford Park, Adelaide, SA 5043 Australia; 3 Istituto Auxologico Italiano IRCSS, Sleep Medicine Center, Department of Cardiology, San Luca Hospital, Milano, Italy; 4 Department of Medicine and Surgery, University of Milano-Bicocca, Milan, Italy; 5 Respiratory Medicine and Sleep Laboratory, Department of Experimental and Clinical Sciences, University of Brescia and Spedali Civili, Brescia, Italy; 6 Department of Internal Medicine, Spedali Civili, Brescia, Italy; 7 Monash Lung and Sleep, Monash Medical Centre, Clayton, VIC Australia; 8 School of Clinical Sciences, Monash University, Melbourne, VIC Australia; 9 Monash Partners – Epworth, Victoria, Australia; 10 Maugeri Institute IRCCS, Sleep Medicine Center, Pavia, Italy; 11 School of Information Technology and Electrical Engineering, The University of Queensland, Brisbane, Australia; 12 University of California San Diego, La Jolla, CA USA; 13 Department of Allergy Immunology and Respiratory Medicine and Central Clinical School, The Alfred and Monash University, Melbourne, Australia

**Keywords:** Desaturation, Hypoxia, Chemosensitivity, Dyspnea, Prognosis

## Abstract

**Background:**

Despite considerable progress, it remains unclear why some patients admitted for COVID-19 develop adverse outcomes while others recover spontaneously. Clues may lie with the predisposition to hypoxemia or unexpected absence of dyspnea (‘silent hypoxemia’) in some patients who later develop respiratory failure. Using a recently-validated breath-holding technique, we sought to test the hypothesis that gas exchange and ventilatory control deficits observed at admission are associated with subsequent adverse COVID-19 outcomes (composite primary outcome: non-invasive ventilatory support, intensive care admission, or death).

**Methods:**

Patients with COVID-19 (*N* = 50) performed breath-holds to obtain measurements reflecting the predisposition to oxygen desaturation (*mean desaturation* after 20-s) and reduced chemosensitivity to hypoxic-hypercapnia (including *maximal breath-hold duration*). Associations with the primary composite outcome were modeled adjusting for baseline oxygen saturation, obesity, sex, age, and prior cardiovascular disease. Healthy controls (*N* = 23) provided a normative comparison.

**Results:**

The adverse composite outcome (observed in *N* = 11/50) was associated with breath-holding measures at admission (likelihood ratio test, *p* = 0.020); specifically, greater *mean desaturation* (12-fold greater odds of adverse composite outcome with 4% compared with 2% desaturation, *p* = 0.002) and greater *maximal breath-holding duration* (2.7-fold greater odds per 10-s increase, *p* = 0.036). COVID-19 patients who did not develop the adverse composite outcome had similar *mean desaturation* to healthy controls.

**Conclusions:**

Breath-holding offers a novel method to identify patients with high risk of respiratory failure in COVID-19. Greater breath-hold induced desaturation (gas exchange deficit) and greater breath-holding tolerance (ventilatory control deficit) may be independent harbingers of progression to severe disease.

**Supplementary Information:**

The online version contains supplementary material available at 10.1186/s13054-021-03630-5.

## Background

COVID-19 outcomes are notoriously unpredictable [1]: roughly 5% of patients exhibit severe and progressive pneumonia that requires intensive care in the form of mechanical ventilatory support [2]. Favorable outcomes of serious cases rely on judicious administration of ventilatory support for those likely to benefit most [3], but such resources have become uniquely limited during peaks in the pandemic. While known risk factors for adverse outcomes include older age [2, 4–7], lower baseline pulse oxygen saturation (SpO_2_) [5, 7, 8], obesity [4], cardiovascular comorbidities [4–6], and inflammatory biomarkers (e.g. C-reactive protein [6]), considerable unexplained heterogeneity remains [5, 9]. A deeper understanding of why some patients deteriorate after admission while others recover with minimal intervention is needed. Such knowledge could help facilitate earlier administration and prioritization of advanced emerging medical interventions.

One of the most consistent risk factors for adverse outcomes of COVID-19 is lower baseline SpO_2_ [5, 7, 8], a reflection of disease-related gas exchange deficits (e.g. ventilation/perfusion [V/Q] heterogeneity). In addition to baseline SpO_2_, reports from Italy early in the pandemic described successful triage of patients using exertional desaturation (cardiometabolic challenge) [10–12] as a means to reveal gas exchange abnormalities. However, the role of exertional desaturation as a risk factor independent of baseline SpO_2_ remains unproven [12, 13]. Ground glass opacities and consolidation seen in computed tomography prior to respiratory failure [5, 14, 15] suggest that gas exchange deficits are a likely risk factor. Yet, to date, the propensity for additional rapid desaturation with a ventilatory challenge (breath-holding) has not been examined as a risk factor. In principle, breath-holding is expected to yield rapid desaturation in those with early gas exchange deficits (V/Q heterogeneity and reduced functional lung gas volumes [16–18]) beyond baseline SpO_2_. Moreover, in the context of anecdotal reports of “silent hypoxemia” (disproportionate tolerance of hypoxemia) as a characteristic of COVID-19 [19, 20], we and others [21, 22] considered that blunted ventilatory control (reduced chemosensitivity) may be an adverse neurophysiological consequence of infection, and could feasibly predispose to respiratory failure. By contrast, others have suggested that a robust ventilatory responses may promote patient self-inflicted lung injury (P-SILI) [23, 24], although this notion remains contentious [25]. There is a lack of available physiological data on the risks of respiratory failure associated with blunted *v.* robust ventilatory control in patients with COVID-19.

Accordingly, in a prospective, multi-center, observational study, we aimed to determine whether gas exchange and ventilatory control deficits in patients admitted for COVID-19 are associated with adverse outcomes of the disease (primary composite outcome of non-invasive pressure support, intensive care admission, or death). We used a simple, non-invasive, recently-validated breath-holding technique [26] to test the hypothesis that adverse outcomes are independently associated with (1) greater *mean desaturation* during a fixed-duration (20-s) breath-hold*,* and (2) reduced chemosensitivity based on greater maximal breath-hold duration [26–28]. The magnitude of the spontaneous ventilatory response following 20-s breath-holds (lower in those with reduced chemoreflex sensitivity) was also evaluated as a risk factor [26].

## Methods

### Participant recruitment

Fifty-seven hospitalized patients aged 18–90 year were enrolled after clinical diagnosis of COVID-19 in three different centers in northern Italy (Brescia, Milan, Pavia). Diagnosis was confirmed with a positive nasal or pharyngeal swab or with clear clinical evidence (i.e. typical signs at laboratory blood tests and computed tomography and/or chest ultrasound) when the swab result was yet not available (swab positivity was confirmed in all patients eventually). Exclusion criteria were: more-than-moderate dyspnea (Borg ≥ 4), hemodynamic instability, Brescia-COVID respiratory severity scale > 1 [29], diurnal home treatment with supplemental oxygen or ventilatory support, use of sedatives, opioids, anti-emetics or other drugs known to impact chemosensitivity, heart failure, chronic obstructive pulmonary disease, pregnancy, and inability to understand the informed consent.

Twenty-four healthy controls were contemporaneously studied in Melbourne (Monash Health), which at the time had low case rates of COVID-19. Absence of COVID-19 was assessed by medical examination. Exclusion criteria also included professional divers, singers, or trumpeters.

### Breath-holding procedure and analysis

In patients, tests were performed shortly after admission, while breathing room air.

Breath-holding maneuvers were performed as described previously [26]. Briefly, while supine, participants were instructed to breathe only through the nose, hold their breath starting from residual functional capacity (FRC) and avoid deep inspiration prior to breath-holds. Ambulatory equipment designed for diagnosis of sleep apnea was used: nasal flow was recorded via an uncalibrated nasal cannula, together with digital saturation recorded via a probe with signal averaging time of 3-s or faster (8000J, Nonin, Plymouth, MN). Investigators requested ≥ 4 reliable *20-s fixed-time breath-holds* and ≥ 1 *maximal breath-hold* (up to 90 s). Additional details are provided in Additional file 1.

**Fig. 1:**
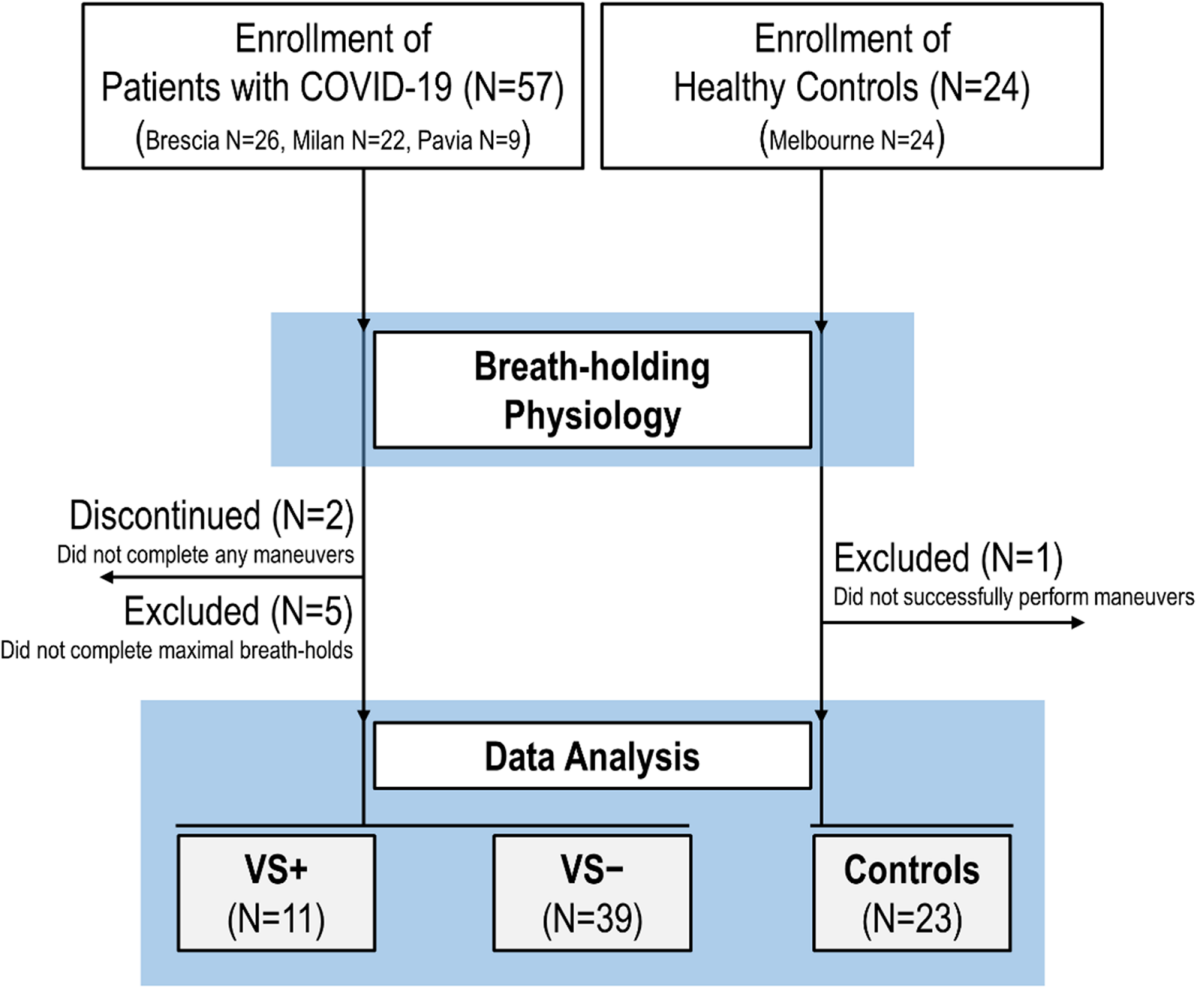
Participant flow diagram. Due to discomfort related to the procedures, *N* = 2 COVID-19 patients aborted the study before completing any breath-hold, *N* = 5 did not complete maximal breath-holds, leaving 50/57 with available data. *N* = 1 control could not complete any breath-holding. We note that (exploratory) re-inclusion of the *N* = 5 patients who had available mean desaturation data (but not maximal duration variables) had no meaningful impact on the associations between mean desaturation and adverse outcomes. Patients who met the criteria for the adverse primary composite outcome are denoted “VS + ” (*N* = 4 non-invasive bi-level pressure support, *N* = 7 intensive care, *N* = 1 death; “VS − “ indicates patients discharged without meeting adverse primary outcome criteria

Three physiological measurements were calculated (custom MATLAB software): *mean desaturation* (change from baseline in SpO_2_ after 20-s of apnea, using ensemble averaging and delay-correction), *maximal breath-hold duration* (largest value observed) [26], and *ventilatory response* (ventilatory overshoot at the second recovery breath following the 20-s breath-hold; ensemble-averaged tidal volume × rate; percent of pre-breath-hold baseline [26]). Participant flow chart is illustrated in Fig. 1.

### Statistical analysis

The adverse composite outcome was reached with any occurrence of non-invasive bi-level pressure support, intensive care admission, or death. Since patients with COVID-19 who met the criteria for the adverse primary outcome ultimately received ventilatory support (non-invasive or via intubation in intensive care), we refer to these individuals herein as VS +; patients discharged without experiencing the adverse primary outcome were labelled VS − (Fig. 1).

The primary hypothesis was quantified by comparing the fully adjusted model against a reference model without breath-holding measurements using a likelihood ratio test. Significance of this single test (*p* < 0.05) was taken to indicate that the 3 measurements (together) explained unique heterogeneity in the primary adverse outcome after that already explained by 5 primary model covariates (baseline SpO_2_, BMI, age, sex, cardiovascular disease). Subsequent analysis then examined associations with individual breath-holding measures.

Additional serial modeling analysis examined associations between the adverse composite outcome and individual breath-holding measures, with progressive adjustment for other breath-holding measures and covariates. Multivariable linear regression models also examined differences in breath-holding measures between VS +, VS −, and controls, adjusting for covariates. Baseline SpO_2_ and mean desaturation were considered confounders in the analysis of associations between outcomes and breath-holding duration (i.e. greater desaturation was considered a confounding source of shorter breath-hold duration independent of chemosensitivity [30]).

A parsimonious model was also developed (removed covariates with high uncertainty per *p* > 0.2) to reduce uncertainty in the remaining model coefficients.

To illustrate that the knowledge provided could potentially help with the development of a future prediction tool, we assessed the preliminary prognostic value (discriminatory capacity) of the parsimonious model, understanding the limitations of the small dataset for this purpose. Accuracy was examined with versus without the breath-holding measures (random perturbation analysis and cross-validation).

Further details, including a priori power analysis to support the sample size, are available in the Additional file 1.

### Role of the funding source

This study was not directly funded.

## Results

Of those enrolled, 50/57 patients with COVID-19 and 23/24 non-COVID controls completed testing and provided data for analysis (Additional file 1). Breath-holding tests were performed on the day of enrolment. Baseline characteristics of the analyzed participants are shown in Table 1. Overall, *N* = 11/50 patients with COVID-19 met the primary composite outcome criteria (ventilatory support, intensive care, or death).

**Table 1:** Baseline characteristics

Characteristic	COVID-19 patients			Controls (*N* = 23)
	All (*N* = 50)	VS + (*N* = 11)	VS − (*N* = 39)	
Population factors				
Age, years	59.6 ± 13.6	62.7 ± 7.5	58.8 ± 15.0	45.4 ± 10.6
Female sex, *n* (%)	8 (16)	0 (0.0)	8 (20.5)	14 (60.9)
Body mass index, kg/m^2^	26.4 ± 4.0	28.1 ± 4.2	25.9 ± 3.4	25.2 ± 4.7
Caucasian or white race/ethnicity, *n* (%)	44 (88)	10 (90.9)	34 (87.2)	19 (82.6)
History				
History of hypertension, *n* (%)	26 (52)	9 (81.8)	17 (43.6)	1 (4.3)
History of cardiovascular disease, *n* (%)	4 (8)	1 (9.1)	3 (7.7)	0 (0.0)
History of diabetes, *n* (%)	8 (16)	2 (18.2)	6 (15.4)	0 (0.0)
Current smoking, *n* (%)	6 (12)	3 (27.3)	3 (7.7)	0 (0.0)
Current medications				
*β*-blockers, *n* (%)	4 (8)	1 (9.1)	3 (7.7)	0 (0.0)
ACE-inhibitors, *n* (%)	9 (18)	4 (36.4)	5 (12.2)	0 (0.0)
ARB, *n* (%)	4 (8)	2 (18.2)	2 (5.1)	0 (0.0)
Clinical presentation at admission				
Baseline SpO_2_, %	94.5 ± 4.6	89.9 ± 6.6	95.8 ± 2.3	97.6 ± 1.0
PaO_2_ at admission, mmHg*	76.2 ± 15.3	71.0 ± 16.4	77.7 ± 14.9	–
PaCO_2_ at admission, mmHg*	36.4 ± 5.4	37.0 ± 4.6	36.2 ± 6.0	–
Baseline Heart rate, beats/min	86.8 ± 16.8	86.2 ± 21.0	86.9 ± 13.6	–
Baseline systolic blood pressure, mmHg	127.3 ± 19.1	126.5 ± 18.8	127.5 ± 19.8	–
Baseline diastolic blood pressure, mmHg	78.5 ± 10.8	74.4 ± 12.5	79.7 ± 10.2	–
Anosmia, *n* (%)	11 (22.0)	1 (9.1)	10 (25.6)	–
Ageusia, *n* (%)	12 (24.0)	2 (18.2)	10 (25.6)	–
Gastrointestinal symptoms, *n* (%)	13 (26.0)	2 (18.2)	11 (28.2)	–
Dyspnea presence, *n* (%)	13 (26.0)	3 (27.3)	10 (25.6)	–
Laboratory tests				
C-reactive protein, mg/L	20.2 ± 41.9	14.6 ± 23.7	21.8 ± 15.0	–
D-dimer, μg/L	0.8 ± 0.8	1.1 ± 0.8	0.7 ± 0.9	–
Breath-holding measurements				
Mean desaturation, %	2.34 ± 1.78	4.42 ± 2.37	2.21 ± 1.43	1.57 ± 1.24
Maximal breath-hold duration**, s	46.8 ± 16.9	51.8 ± 12.7	41.8 ± 14.2	53.0 ± 20.3
Recovery breath, %_baseline_	188.9 ± 61.3	184.6 ± 81.8	195.0 ± 62.1	180.5 ± 49.4
Outcomes				
Discharge:oxygen:ventilatory support, *n*	14:36:11	0:11:11	14:25:0	–
Duration of hospitalization, days	29.8 ± 17.2	47.4 ± 12.5	24.8 ± 15.3	–

Data are expressed as mean ± SD or as *N* (%). Patients who met the criteria for the adverse primary composite outcome are denoted “VS + ” (*N* = 4 non-invasive bi-level pressure support, *N* = 7 intensive care, *N* = 1 death). In all patients, diagnosis was confirmed with a positive nasal or pharyngeal swab. All patients admitted to ICU were administered mechanical ventilatory support; the patient who died was also in intensive care on mechanical ventilatory support. “VS − “ indicates patients discharged without meeting adverse primary outcome criteria. Average time to the primary outcome in VS + patients was 6  [2] days (median [IQR]). Non-Caucasian/Non-White race/ethnicities were black (1 VS + and 1 VS − COVID-19 patients), Hispanic (3 VS + COVID-19 patients) and Asian (1 VS − COVID-19 patient and 4 controls). Note that participants included in the table are those who provided data for all breath-holding measures. “Discharge” indicates discharge without oxygen or interventions that met criteria for the primary outcome during the hospital stay. On average, 3.8 ± 1.0 20-s breath-holds per individual were analyzed to calculate the ventilatory response variable (3.6 ± 0.9 in controls); 1.6 ± 0.6 maximal breath-holds were used to determine maximal breath-hold duration (2.1 ± 0.5 in controls); 5.2 ± 1.1 breath-holds (20-s or maximal) were used to calculate mean desaturation (6.3 ± 1.1 in controls). *Data available for 42 patients (9 VS + and 33 VS −). *Abbreviations:* SpO_2_, peripheral oxyhemoglobin saturation; PaO_2_, partial pressure of oxygen; PaCO_2_, partial pressure of carbon dioxide; ACE, angiotensin converting enzyme; ARB, angiotensin II receptor blockers. **Data shown are adjusted for baseline SpO_2_ and mean desaturation (regression)

### Primary outcome assessment

#### Pre-specified primary analysis

Within COVID-19, breath-holding variables explained unique heterogeneity in the primary composite outcome adjusting for covariates (likelihood ratio 0.0073, *p* = 0.02 *v.* model without breath-holding measures; see Table 2). Specifically: (1) the odds of the composite outcome increased 3.6-fold per 1%_Hb_ greater *mean desaturation* (log-odds [*β* ± SEM] = 1.27 ± 0.59 per %_Hb_, *p* = 0.002). (2) A 10-s longer *maximal breath-hold duration* raised odds of the primary outcome 2.7-fold (log-odds = 0.10 ± 0.05 per sec, *p* = 0.037). Note these odds are adjusted for baseline SpO_2_ and other covariates. However, the *ventilatory response* variable was not associated with the primary outcome. Significance was not altered by exploratory inclusion of additional available covariates to the reference model (e.g. C-reactive protein, d-dimer, hemoglobin) or omission of existing covariates (Additional file 1).

**Table 2:** Association between breath-holding measurements and adverse outcomes of COVID-19

	Primary model *β* ± SEM (*p* value)	Parsimonious model *β* ± SEM (*p* value)
Breath-holding measurements		
Mean desaturation (%Hb)	1.27 ± 0.59 (0.002)	1.25 ± 0.54 (0.001)
Maximal breath-hold duration (s)	0.10 ± 0.05 (0.037)	0.10 ± 0.05 (0.020)
Ventilatory response (%_baseline_)	0.00 ± 0.01 (0.9)	—
Covariates		
Body mass index (kg/m^2^)	0.22 ± 0.22 (0.3)	0.27 ± 0.19 (0.157)
Baseline SpO_2_ (%)	− 0.44 ± 0.22 (0.001)	-0.45 ± 0.22 (0.001)
Cardiovascular disease	4.47 ± 2.71 (0.064)	5.27 ± 2.38 (0.019)

**Fig. 2:**
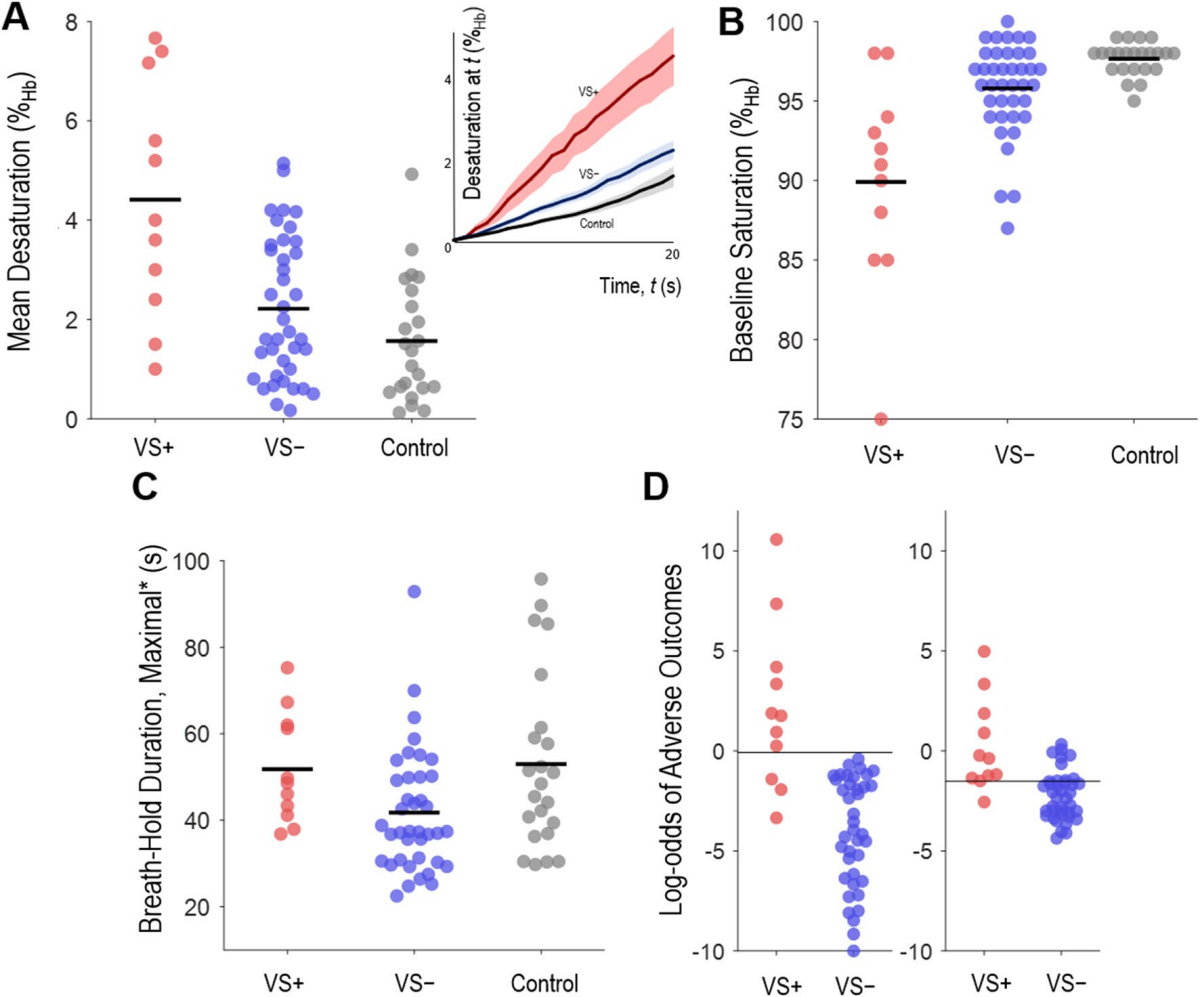
Individual breath-holding measurements in COVID-19 patients who experienced the adverse primary composite outcome (and required ventilatory support “VS + ”, *N* = 11), in COVID-19 patients without adverse outcomes (“VS − “, N = 39), and controls (*N* = 23). Horizontal black bars overlying individual data indicate group mean values. **A**. Mean desaturation after 20-s, unadjusted. Inset: desaturation profile for each group (mean ± SEM desaturation at any time, delay corrected). **B**. Baseline oxygen saturation, a potential confounder for mean desaturation, was different between groups and independently associated with adverse COVID-19 outcomes. **C**. Maximal breath-hold duration (*shown adjusted for baseline SpO_2_ [+ 2.6 s per %_Hb_ below 97.6] and mean desaturation [+ 0.6 s per %_Hb_ above 1.57]); shorter adjusted durations were interpreted as increased chemosensitivity (or sensitivity to dyspnea). Data on ventilatory response to breath-holds (not shown) were similar between groups. **D**. Left: Multivariable logistic regression model output for each individual patient (parsimonious model, Table 2) shows that mean desaturation and maximal breath-hold duration (+ covariates baseline SpO_2_, body mass index, cardiovascular disease) provides good outcome discrimination (threshold ~ 0); a score of 0 represents 50% probability of the adverse primary outcome. Each 1-point increase represents a log (2.7-fold) increase in likelihood of the primary outcome. Right: Reference model without breath-holding measures (baseline SpO_2_, body mass index, cardiovascular disease) showed significantly reduced outcome discrimination (*p* = 0.007, random permutation analysis)

Association between breath-holding measurements at admission and the adverse composite primary outcome in COVID-19 (multivariable logistic regression). Data shown are *β* ± SEM (*p* value); *β* describes the increase in log-odds of the adverse outcome per change in exposure variable. Primary model: The breath-holding measurements significantly improved the model (likelihood ratio 0.0073, *p* = 0.02) over a reference model with covariates only (body mass index, baseline SpO_2_, cardiovascular disease [1 = Present, 0 = Absent], plus age and sex [not shown]). *p* values are based on likelihood ratio tests. The parsimonious model is a simplified and refined version of the primary model (age, sex, and ventilatory response were removed [*p* > 0.2]; Intercept = 25.31 ± 17.31). The potential predictive value of the model is illustrated in Fig. 2D and a tool for risk calculation is provided in Additional file 2

#### Sensitivity analysis

Greater *mean desaturation* raised the odds of the adverse composite outcome in simple bivariate analysis (log-odds of VS +: 0.67 ± 0.23 per %_Hb_, *p* = 0.0007), when additionally adjusted for aforementioned covariates including baseline SpO_2_ (0.65 ± 0.33, *p* = 0.023), and with further adjustment for maximal breath-hold duration (1.26 ± 0.56, *p* = 0.002). By contrast, *maximal breath-hold duration* was only associated with the primary outcome with (but not without) the appropriate adjustment for *mean desaturation* (Table 2).

### Group differences

In adjusted analysis, VS + patients (*N* = 11) exhibited greater *mean desaturation v.* VS − patients (*N* = 39; difference[95%CI] 1.6[0.3–2.8]%_Hb_, *p* = 0.009) and controls (*N* = 23; 2.3[0.8–3.9]%_Hb_, *p* = 0.002; Fig. 2A); differences were clear despite adjustment for the lower baseline SpO_2_ seen in VS + (versus VS −: 5.8[3.5–8.0] %_Hb_, versus controls: 7.5[4.7–10.3]%_Hb_, *p* < 0.0001, see Fig. 1B). VS − patients, but not VS + patients, had shorter adjusted maximal breath-hold duration than controls (difference = 15.9[5.6–26.1] s, *p* = 0.002, Fig. 2C).

### Potential for outcome discrimination in COVID-19

The parsimonious model (Table 2) describing risks associated with *mean desaturation* and *maximal breath-hold duration* exhibited discriminative potential (Fig. 2D-*left*): model accuracy was 94[88–100]% (*p* < 0.0001 *v.* chance), which was significantly superior (random permutation analysis, *p* = 0.007) to a reference model (covariates only; includes baseline SpO_2_) without breath-holding measurements (reference accuracy = 80[69–91]%, Fig. 2D-*right*). The breath-holding model—but not the reference model—passed cross validation analysis (accuracy 80[69–91]%, *p* = 0.003; reference model accuracy 70[57–83]%, *p* = 0.24).

### Relationship to dyspnea

Given associations between adverse outcomes and greater breath-holding tolerance (maximal breath-hold duration) we performed additional analysis of dyspnea at admission: In fully adjusted analysis, dyspnea (Borg 1–3 *v.* Borg = 0) was also associated with *reduced* risk of the primary outcome in COVID-19 in the current sample ([log-odds: − 2.8 ± 1.6, *p* = 0.038). Inclusion of dyspnea in the above models did not meaningfully alter the findings reported above (see Additional file 1).

## Discussion

Our prospective observational study demonstrated that adverse outcomes of COVID-19 (composite outcome of non-invasive ventilation, intensive care admission, or death) are associated with deficits in gas exchange and ventilatory control revealed using a validated breath-holding technique. Specifically, we demonstrated that with increasing predisposition to oxygen desaturation during breath-holding there is higher risk of progression to severe disease, independent of baseline oxygenation, and other key covariates. Greater maximal breath-hold duration—adjusted for hypoxemia (i.e. baseline saturation)—was also an independent risk factor among patients with COVID-19. Our finding that blunted ventilatory control [26–28] is a deleterious physiological feature of COVID-19 does not support the notion that vigorous ventilatory drive promotes self-inflicted lung injury (P-SILI) [31–33], but rather is consistent with “silent hypoxemia” as a prevalent characteristic in COVID-19 [13–15, 34, 35]. A third breath-holding variable, the ventilatory response to 20-s breath holds, was not associated with the primary outcome. Each analysis demonstrated that associations between breath-holding variables and the adverse outcomes (as well as outcome prediction) were seen above and beyond baseline saturation (and other commonly-available clinical measures). Overall, the current study provides unique insight into the differential physiological characteristics of patients who exhibit adverse outcomes *v.* those who do not. We consider that this knowledge has the potential to be used in future tools to identify patients at elevated risk of adverse outcomes in COVID-19.

### Novel physiological insights

#### Desaturation

Early signs of gas exchange deficits in patients who later develop severe COVID-19—such as regional ventilation/perfusion (V/Q) heterogeneity and reduced lung gas volumes—have been inferred from chest imaging [5, 14, 15]; however, functional evidence of this notion is limited. Here, we show that breath-holding unmasks a greater COVID-19-related decline in gas exchange in those who progress to severe disease versus those who do not. Specifically, after adjusting for baseline SpO_2_, breath-holding desaturation is an independent risk factor for adverse outcomes in COVID-19. Indeed, breath-holding is expected to provide unique information on gas exchange deficits on the basis that V/Q heterogeneity (lower V/Q regions readily desaturate during apnea) [16] and reduced lung gas volumes (greater decline in alveolar PO_2_ per unit time) [17, 18] influence desaturation speed independently of baseline SpO_2_. Of note, baseline SpO_2_ can be insensitive to reduced PaO_2_ when on the plateau of the SpO_2_/PaO_2_ curve. Our findings also withstood adjustment for obesity and pre-existing cardiovascular disease as confounders, suggesting that these particular non-COVID sources of variability do not explain away the associations observed.

#### Ventilatory control

The finding that longer maximal breath-hold duration (SpO_2_-adjusted [30]) confers risk in COVID-19 suggests that blunted ventilatory control responses [26–28] may precede severe versus milder disease. Supporting this notion, absence of dyspnea (Borg = 0) was also an independent risk factor for adverse outcomes in the current study (*n.b.* impaired chemosensitivity may mitigate dyspnea [36–40]). Notably, COVID-19 is associated with anosmia/dysgeusia, and investigators have previously speculated infection at the carotid bodies [21] or centrally [34]. Regardless, it is unclear whether ventilatory control effects of COVID-19 are causally involved in respiratory failure or simply a marker of more severe viral illness. Indeed, interventional studies using ventilatory stimulants could shed light on the putative causal pathways. We also observed that patients who did not develop severe disease had shorter breath-hold durations *v.* healthy controls; thus, such individuals may escape adverse outcomes of COVID-19 partly through a more robust ventilatory control defense against hypoxia/hypercapnia. Overall, our data do not support the concept that robust chemoreflexes exacerbate lung injury via greater chemoreflex-related transpulmonary pressures (P-SILI) [23, 24]. Instead, our study suggests that blunted ventilatory control in the face of hypoxemia (i.e. “silent hypoxemia”) is additionally deleterious in COVID-19 [19, 20, 22, 34, 35]. Nonetheless, our findings suggest that breath-holding duration may be a clinically important biomarker for identifying risk of subsequent respiratory failure regardless of the underlying mechanisms.

### Clinical implications

Understanding risk factors for adverse outcomes of COVID-19 has been the focus of intense research over the last 12 months. To date, notable studies examining risk factors have been retrospective in design [2, 4–9, 14]. In the current prospective study, greater breath-holding desaturation and reduced maximal breath-holding duration were associated with adverse outcomes in COVID-19 independently of baseline SpO_2_, and early analysis suggests that the approach has potential predictive value. Exploratory inclusion of existing biomarkers C-reactive problem and d-dimer, and adjustments for haemoglobin levels did not change our findings. Our translational work therefore demonstrates the feasibility of using physiological testing to estimate the risk of adverse COVID-19 outcomes days in advance of patient deterioration, enabling prioritization of limited resources to the high risk patients who need them most (Fig. 2D), and providing a window for early administration of medical therapies (e.g. dexamethasone) prior to advanced disease progression, especially in pandemic epicenters (an excel prognostic tool is also provided for academic evaluation). These findings are consistent with anecdotal reports of clinicians in Italy and beyond successfully using exertional test-derived altered SpO_2_ values to triage patients with Covid-19 and hospitalization those with post-exertional greater desaturation [10–12]. However, in contrast to exertional tests (i.e. 6-min walking test), breath-holding does not require increased energy expenditure or cardiac output, and obviates walking and associated bystander/caregiver contamination. Measurement of these simple, novel surrogates capturing pulmonary and chemoreflex risk factors requires minimal inventory (i.e. a means to record oximetry and a timing device) and could feasibly provide a useful means to estimate risks of future deterioration in under-resourced circumstances, should future validation studies support this concept.

### Methodological considerations

This study has several limitations. First, while the sample size provided sufficient power to confirm our primary hypothesis (see Additional file 1: Statistical Analysis), we did not have an additional sample for an independent validation analysis (study is forthcoming). However, random permutation analysis and leave-one-out cross validation provided rigor and reassurance that results were not the trivial consequence of overfitting. Second, the number of covariates (potential confounders) included in our model analyses may raise concerns, yet we emphasize that simulations demonstrated that statistical power was not meaningfully reduced by the inclusion of uncorrelated covariates and that the inclusion or removal of covariates did not strength or weaken the associations. Third, given the narrow race/ethnicity of our study population, we cannot generalize our findings to all potential patient populations globally. In addition, our analysis suggests that breath-holding variables are associated with adverse outcomes independent of sex, but we are unable to conclude whether breath-holding variables are associated with adverse outcomes specifically within men or within women, or if sex-specific models might be needed. A larger study of women and men is needed to address these questions. Fourth, breath-holding required cooperation so patients who necessitated immediate ventilatory support were not studied. Nevertheless, we highlight that people requiring urgent intervention are, by definition, readily triaged and beyond the scope of this work. Fifth, the maximal breath-hold variable, but not the ventilatory response variable, suggested increased chemosensitivity was protective. We emphasize, however, that measurement of the ventilatory response was more complex (requires assumptions of nasal breathing and effective linearization of nasal pressure). Of note, the ventilatory response variable is susceptible to measurement noise in the absence of a sealed oronasal mask and pneumotachometer [26] and thus potentially unreliable. Despite these limitations, we believe our findings are clinically important and deserve further study.

## Conclusions

Breath-holding measurements of susceptibility to rapid desaturation and ventilatory control sensitivity are associated with progression to respiratory failure in COVID-19: greater desaturation during breath-holds (interpreted as greater gas exchange deficit) and longer maximal breath-holds (interpreted as lower chemosensitivity) are independent risk factors. Simplified physiological measures of gas exchange and neurophysiological deficits in COVID-19 may hold utility for future translational use in early triage to scarce health care resources or early administration of medical interventions. Our study also raises the possibility that blunted ventilatory control is a therapeutic target for preventing severe disease in COVID-19, a concept that will require interventional studies to assess.

## Supplementary Information

**Additional file 1**: Supplemental text (methods and results) and tables.

**Additional file 2: **Tool to assess probability of the primary adverse outcome (for academic evaluation).

## Data Availability

All the individual participant and summary data collected during the trial will be shared upon request, after de-identification. Additionally, study protocol, statistical analysis plan and informed consent will be made available. Data will be available immediately following publication and ending 5 years following article publication with researchers who provide a methodologically sound proposal to achieve aims in such approved proposal. Proposals should be directed to LM ludovico.messineo@yahoo.it or EP e.perger@auxologico.it; access will require a data use agreement.
