# Supplementary material for: Breath-holding as a novel approach to risk stratification in COVID-19
Source: Crit Care. 2021 Jun 14;25:208. doi: 10.1186/s13054-021-03630-5 (PMC8200551; doi:10.1186/s13054-021-03630-5)
Supplement: Supplementary file 1 — Additional file 1: Supplemental text (methods and results) and tables. [file 13054_2021_3630_MOESM1_ESM.doc]

**Online Supplement**

**Breath-holding as a novel approach to risk stratification in COVID-19**

Ludovico Messineo, MD*, Elisa Perger, MD*, Luciano Corda, MD, Simon A Joosten, MBBS, PhD, Francesco Fanfulla, MD, Leonardo Pedroni, Philip I Terrill, PhD, Carolina Lombardi, MD, Andrew Wellman, MD, PhD^1^, Garun S Hamilton, MBBS, PhD, Atul Malhotra, MD, Guido Vailati, Gianfranco Parati, MD, Scott A Sands, PhD.

**METHODS**

## Protocol

All recruitment centers were located within 200 m of sea level.

For each Covid-19 patient the following data were collected on admission to the Covid-19 dedicated unit: medical history, medications (including recent previous administration of Covid-19 general treatment such as antivirals, antibiotics, hydroxychloroquine and steroids), baseline demographics (i.e. age, body mass index [BMI], ethnicity), baseline SpO2, heart rate and blood pressure, presence of dyspnea (Borg=1-3 v. Borg=0) or other current symptoms of Covid-19 (anosmia, ageusia, nausea/vomiting, and diarrhea, Y/N), and history of smoking (current, prior, never). Within 24 hours after admission, an arterial blood gas sample and blood tests were recorded where available. Blood tests included measurement of C-reactive protein (CRP) levels, white blood cell count, hemoglobin, lactate dehydrogenase, fibrinogen and d-dimer levels. Subsequently, breath-holds were performed.

Patients were then treated according to the standard medical care. The following outcomes, if present, were recorded: first administration of oxygen therapy, first administration of bi-level PAP, admission to intensive care unit (ICU), death, or discharge. Hospitalization duration was also recorded.

Healthy controls arrived at the laboratory during the day and underwent a routine medical examination. Exclusion of disease and current medication use was confirmed. Baseline data (i.e. age, ethnicity and BMI) were recorded, together with baseline values of pulse oxygen saturation (SpO_2_). History of smoking (current, prior and both) was also annotated. Finally, breath-holds were performed.

All patients and equipment were handled in accordance with WHO recommendation of personal protection in the healthcare setting . Specifically, “dirty” units or “dirty” rooms were dedicated exclusively to these patients and accessed only with full set of personal protective equipment. Reusable items, including laptops and other instruments to record breath-holds, were appropriately disinfected or sterilized before next use and one-time-only use items were appropriately disposed.

## Breath-Holding Procedures

To facilitate breath-holding assessment, oxygen-therapy prescribed upon admission, if present, was temporarily withdrawn.

*Equipment.* Additional measures included thoracoabdominal movements and single-channel electrocardiogram (not used for subsequent data analysis) for quality control and safety purposes. Wherever possible, a movable cart with a laptop for live data recording was brought to the patient’s bedside, otherwise data collection was performed remotely. Two operators supervised the procedure to ensure participant’s comfort, provide coaching on the maneuvers and assess the quality of the recorded signals. Note that if remote control of data acquisition was in place, one of the operators ensured data quality at of the laptop outside of the “dirty” room/unit. Each breath-holding session lasted 15-to-30 min. Data collection was performed with RemLogic 3.4 (Embla systems, Broomfield, CO) and Profusion 4 (Compumedics Limited, Victoria, Australia).

*Procedure.* During each procedure, verbal feedback was provided as needed. During the 20-s fixed-time breath-holds, participants were asked to hold their breath after at least 30-s of quiet breathing and then asked to restart breathing after 20-s of apnea. The procedure was repeated up to six times every two minutes until at least 4 reliable maneuvers were obtained. During the maximal breath-holding procedure, participants were coached (in Italian or in English language) to facilitate a longer duration with verbal encouragement such as: “keep going”, “go, go, go”, “try to resist” (in Italian/English as appropriate), along with feedback on the status of the breath-hold duration every 5-s, according to our previously-validated technique . The procedure was repeated up to three times every two minutes until at least one reliable maneuver was obtained; participants were told their previous breath-hold time to be encouraged to beat the previous record. Maneuvers were repeated (i.e. considered not reliable) if the “live” flow signal was not completely flat for the entire apnea time, if the participant did not start from FRC or if the minimal duration of 20-s was not reached.

## Data Analysis

Data were exported in European Data Format and analyzed in MATLAB (Mathworks, Natick, MA, USA) by a single investigator (LM).

*Mean desaturation*. The “circulatory delay” was calculated for each participant as the time from the end of the breath-holds to the nadir of the (ensemble-averaged) SpO_2_ signal (synchronization at end-apnea) . The SpO_2_ signal was subsequently time-shifted by the estimated circulatory delay, and the time course of desaturation during each breath-hold (start breath-hold was set to zero) was tabulated. Finally, data were ensemble averaged.

To avoid averaging any “re-saturation” data with desaturation data, each data point after time = 0 was modified to be equal to the minimum SpO_2_ value observed up to that time (consequently any SpO_2_ values after the nadir are set to the nadir value).

Note that this type of analysis allowed us to ensemble average data from the 20-s breath holds (i.e. time 0 to end of breath-hold) and maximal breath holds (i.e. time 0 to 20th second of breath hold).

*Ventilatory response*. This biomarker relies on an uncalibrated but quantitative measurement of airflow. Briefly, ventilation data were overlaid (ensemble averaged) and aligned at end-apnea. The second breath, rather than the first, was used to measure the ventilatory overshoot because the first breath is typically noisier and less reliable than the second, based on our previous experience in measuring this variable . Note that modifications from the prior analysis have been made to use uncalibrated ventilation rather than calibrated pneumotach-based ventilation by using linearized and mean-normalized nasal pressure .

## Statistical Analysis

The statistical analysis plan (choice of primary outcome, statistical model and approach, model covariates) was prespecified in the study protocol. Cardiovascular disease was added to the list of *a priori* covariates—baseline SpO_2_, BMI, age, sex—as a meaningful trend was observed between cardiovascular disease and adverse outcomes.

*Primary model analysis: Multivariable logistic regression.* In primary analysis, a single multivariable logistic regression model quantified the associations between the primary adverse composite outcome (dichotomous dependent variable VS+) and the three breath-holding variables (i.e. mean desaturation, maximal breath-hold duration, ventilatory response), adjusting for five covariates: baseline SpO2, body mass index (BMI), age, sex, and presence of cardiovascular disease (single dichotomous variable). To provide a single test of whether any or all of the three breath-holding variables (i.e. mean desaturation, maximal breath-hold duration, ventilatory response) explain unique heterogeneity in the dichotomous primary outcome, a likelihood ratio test compared this model against a reference model with covariates only.

*Exploratory adjustment for additional covariates.* We considered the possibility that, after adjusting for an additional measured covariate (below), the breath holding variables may no longer explain unique heterogeneity in the primary outcome. Thus, in exploratory analysis, we further explored whether breath-holding variables continued to be associated with the primary outcome after additional inclusion of several candidate variables, namely: study site, ethnicity (Caucasian vs. non-Caucasian), mean arterial blood pressure, baseline heart rate, presence vs. absence of diabetes, CRP levels, hemoglobin levels, d-dimer levels, and dyspnea (Borg 1-3 vs. Borg=0; note Borg>3 were excluded). Analysis was performed through inclusion of each single additional covariate to the fully adjusted model above (then removed again), one at a time.

*Group comparisons*. Individual breath holding variables were also compared against non-Covid controls to establish normalcy. Comparison to non-Covid controls served to illustrate, mechanistically, that greater mean desaturation in those with ventilatory support (VS+) *v.* those without (VS−) appears due to especially great mean desaturation in VS+ (rather than especially small mean desaturation in VS−). Likewise, comparison to controls also served to illustrate that longer (adjusted) breath-holding duration in VS+ *v.* VS− appears not to be due to especially long durations in VS+ but rather shorter durations in VS−. Multivariable linear regression quantified the association between each biomarker (separately) with Covid-19 outcomes versus controls (Covid-19 VS+ *v.* control, Covid-19 VS− *v.* control) in fully adjusted models. Additional analysis of breath-holding variables further adjusted for mean desaturation during breath-holding, to comprehensively account for confounding influence of hypoxemia (both baseline and during breath-holding) in group differences, as appropriate mechanistically for interpretation of groups differences as indirect surrogates of chemosensitivity.

*Analysis of potential prognostic value*. A parsimonious model was developed to 1) increase confidence in the remaining model coefficients, and 2) provide a simpler model for mechanistic interpretation and future validation. From the fully-adjusted primary model, additional covariates were made available for inclusion (baseline mean arterial blood pressure, baseline heart rate, presence of diabetes, ethnicity) and parameters (covariates and breath-holding variables) were progressively removed (backward elimination) if they did not improve the model (per P<0.2, likelihood ratio tests). Terms were eliminated to increase confidence in the remaining model coefficients and to provide a simpler (parsimonious) model for potential future clinical use. Classification *accuracy* (percentage of correct classifications) was used as a single primary measure of prognostic value (model output cutoff/threshold was selected by optimizing sensitivity plus specificity). Standard errors and comparison to chance accuracy values were based on the normal approximation to the binomial distribution method. Random perturbation analysis compared the measured accuracy versus values obtained with randomized/shuffled biomarker data (5000 iterations) to determine statistical significance. Accuracy was also reassessed using leave-one-out cross validation (including entire parameter selection process) for an unbiased representation of future performance on unseen patients.

*Power analysis* (per simulations of primary logistic regression analysis) estimated that 50 patients (20% incidence of primary outcome, three breath-holding variables, five uncorrelated covariates) would provide 90% power to detect associations driven by a 1.1 SD difference between groups in one of three breath-holding variables (alpha = 0.05); the minimum detectable difference was lower (1.0 SD) for planned N=100 patients. Power was not meaningfully affected by the addition of a sixth uncorrelated covariate (see *Exploratory adjustment for additional covariates*).

MATLAB (Mathworks, Natick, MA) was used to perform all statistical analyses.

## Statistical Analysis Rationale

*Dichotomous outcome analysis.* A dichotomous primary outcome variable (logistic regression modeling) was selected over time-to-outcome analysis (Cox proportional hazards modeling) because whether or not a patient eventually deteriorates was considered more clinically relevant than the particular day of deterioration. Specifically, whether a patient can be sent home versus needs closer monitoring in times/places of limited resources requires a yes-or-no answer at the time of assessment. We also considered that exact day of deterioration may be more dependent on timing of resource availability than whether or not an individual was ultimately administered ventilatory support or admitted to ICU. For precedent, we cite the first outcomes of Covid-19 analysis presented in Lancet that used multivariable logistic regression. This decision was pre-specified in our statistical analysis plan.

*Adjustment.* Baseline SpO­_2_ and obesity and are known confounders in the assessment of breath-holding desaturation rate. Thus, our analyses of mean desaturation sought to control for baseline SpO­_2_ and BMI. We also acknowledge that hypoxemia may confound the interpretation of chemosensitivity with respect to breath-holding duration. Thus our preferred analysis of breath-holding duration are adjusted for breath-holding mean desaturation in addition to baseline SpO­_2_  and obesity (e.g. primary model). Age and male sex may also influence breath-holding desaturation rates; age in particular is known to influence adverse outcomes of Covid-19 ; thus these variables were also chosen as model covariates.

## Maintenance of Scientific Rigor in Analysis

Prior to any biomarker analysis, all decisions regarding eligibility of participants for analysis (review of protocol completion) and primary outcome variable coding were confirmed and the outcomes database was locked. Of note, no patient who completed all maneuvers was excluded for analysis. After all outcomes and covariates were locked, a single investigator (LM) ran customized semi-automated analysis code using tagged start and end times of breath-holds to auto-generate the 3 breath-holding variables (outcomes data and covariates hidden were hidden during this analysis phase). Once biomarker data were completed, results were exported to the study database, and the updated database was again locked for the statistical analysis phase.

We emphasize that desaturation magnitudes were automatically evaluated, leaving minimal room for any investigator bias. Moreover, breath-holding duration results and overshoot results were not as expected at study initiation (i.e. longer durations and lower overshoot in Covid-19 VS+ *v.* controls were expected), consistent with our goal of minimizing potential investigator bias.

# RESULTS

On average, 3.8 ± 1.0 (mean ± SD) 20-s breath-holds per individual were analyzed to calculate the ventilatory response biomarker (3.6 ± 0.9 in controls); 1.6 ± 0.6 maximal breath-holds were used to determine maximal breath-hold duration (2.1 ± 0.5 in controls); 5.2 ± 1.1 breath-holds (20-s or maximal) were used to calculate the mean desaturation biomarker (6.3 ± 1.1 in controls).

Overall, the maneuvers were well tolerated. One individual (no history of epilepsy) experienced a major seizure during the recording period of the procedure that we considered unlikely related to breath-holding. He was hospitalized for probable syncope the month prior the study) experienced a major seizure during the recording period of the procedure. In analysis of relatedness, we considered 1) the seizure did not happen while the patient was holding their breath or immediately thereafter, and 2) we have previously observed seizures in Covid-19 patients attributed to the viral infection. The event was therefore considered “possibly-related” to the procedure.

Exploratory adjustment of the primary multivariable logistic regression model for additional confounders did not alter the primary findings: study site (likelihood ratio test for presence *v.* absence of biomarkers: P=0.015), mean arterial blood pressure (P=0.028), baseline heart rate (P=0.017), race/ethnicity (P=0.023), presence of diabetes (P=0.020), levels of CRP (P=0.016), hemoglobin levels (P=0.030), d-dimer (P<0.001), or presence of mild-to-moderate dyspnea (Borg>0, P=0.001).

.

## Serial Modeling Analysis of Mean Desaturation

*Group comparisons:* Unadjusted group differences (per multivariable linear regression) illustrated that mean desaturation was similar in VS− patients *v.* controls (difference=0.65±0.41%_Hb_, P=0.12) but were greater in VS+ patients *v.* controls (difference=2.85±0.57%_Hb_, P=0.000004); greater desaturation was also evident comparing VS+ patients *v.* VS− patients (difference=2.20±0.53%_Hb_, P=0.00006). Adjusting for covariates had little impact on the unadjusted group differences (baseline SpO_2_, BMI, sex, age, cardiovascular disease) as described in the manuscript.

## Serial Modeling Analysis of Breath-Hold Duration

*Group comparisons:* Simple group differences (multivariable linear regression) in maximal breath-hold duration illustrated that breath-hold durations were lower in VS− patients *v.* controls (difference=−14.0±4.4 s, P=0.002) but were also lower in VS+ patients *v.* controls (difference=13.3±6.1 s, P=0.03); the latter effect potentially confounded by the greater mean desaturation experienced in VS+ patients (see Figure S1A for unadjusted comparisons). Adjusting for covariates yielded similar results (VS− patients *v.* controls: difference=15.9±5.2 s, P=0.002 as described in the main manuscript; VS+ patients *v.* controls: difference=11.4±8.6 s, P=0.19). After the planned correction for mean desaturation, with concurrent adjustment for covariates, the shorter breath-hold durations in VS− patients *v.* controls remained (Δ=13.9±5.2 s, P=0.006) but the difference between VS+ patients *v.* controls disappeared (difference=−5.4±9.0 s, P=0.5). From this model, individual breath-hold durations adjusted for mean desaturation and baseline SpO_2_ were calculated and plotted for illustrative purposes (see Figure 2C main manuscript, also shown in Figure S1B to facilitate comparison to unadjusted values in Figure S1A).

## Serial Modeling Analysis of Ventilatory Response

No analysis of ventilatory response yielded associations with the primary composite outcome or group differences.

## Modelling of Covid-19 Diagnosis

The three breath-holding variables (together) were also associated with the presence *v.* absence of Covid-19 (likelihood ratio 0.0112, P=0.029), adjusting for primary covariates; shorter maximal breath-hold duration was associated with Covid-19 status.

## Hospitalization time and requirement for oxygen therapy

Breath-holding variables were not significantly associated with hospitalization time or requirements for oxygen therapy.


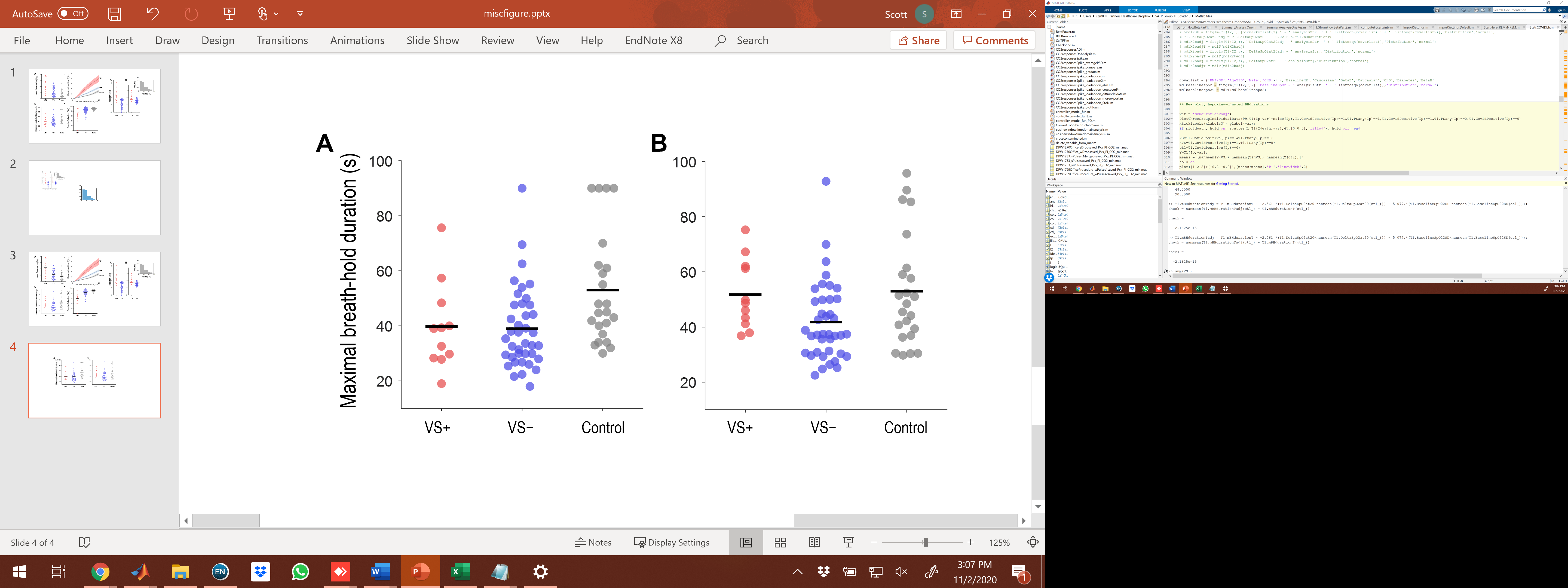


Figure S1. Individual maximal breath-hold duration data before (A) and after (B) adjustment for mean desaturation and baseline oxygen saturation. Groups: VS+ denotes Covid-19 patients who experienced the adverse primary composite outcome (N=11), VS− denotes Covid-19 patients without adverse outcomes (N=39); controls are also shown (N=23). After accounting for lower baseline oxygen saturation and greater mean desaturation (during fixed 20-s breath-holds) in VS+ *v.* other groups (see text), maximal breath-hold durations was no longer reduced in VS+ versus controls (compare B vs A) intepreted as an absence of differences in chemosensitivity. Both before and after adjustment, VS- patients exhibited shorter breath-hold durations that controls. Horizontal black bars overlying individual data indicate mean group values. To adjust for mean desaturation and baseline SpO_2_: model coefficients for the multivariable linear regression model describing breath-hold duration as a function of mean desaturation and baseline SpO_2_, adjusting for other covariates (BMI, sex, age, cardiovascular diseases, VS+ and VS− status) were calculated (coefficients: β±SEM = −2.6±1.3 and 0.6±0.6 s/%_Hb_ respectively); mean desaturation and baseline SpO_2_ were mean-centered (using control data) and combined with the coefficients to subtract out confounding effects of hypoxemia on breath-hold duration.

**REFERENCES**
